# A Case Report of a Primary Conjunctival Diffuse Large B-Cell Lymphoma: Keeping an Eye out Following a Nasopharyngeal Carcinoma

**DOI:** 10.7759/cureus.13904

**Published:** 2021-03-15

**Authors:** Lyna Haybout, Siham Hamaz, Houda Bachir, Habiba Alaoui, Khalid Serraj

**Affiliations:** 1 Internal Medicine, Immunohematology and Cellular Therapy Laboratory, Medical School of Oujda, Mohammed First University, Oujda, MAR

**Keywords:** ocular adnexal lymphoma, non-hodgkin lymphoma, systemic chemotherapy, ucnt

## Abstract

Primary diffuse large B cell lymphoma of the conjunctiva is a rare disease. In this article, we report the case of a 40-year-old man who had previously been treated with chemotherapy and radiotherapy for undifferentiated carcinoma of nasopharyngeal type (UCNT) and who subsequently developed conjunctival lymphoma. We underline through this observation the importance of thinking about a secondary cancer post-radio-chemotherapy even when the clinical presentation is atypical.

## Introduction

Lymphoma of the eyelid is a rare disease (3% of conjunctival tumors), its malignant subtype; diffuse large B-cell lymphoma involving the conjunctiva is in most cases primary and constitutes 3% of conjunctival tumors. We present the case of a man, who had a history of undifferentiated carcinoma of nasopharyngeal type (UCNT), which was treated with chemotherapy and radiation, and in whom conjunctival lymphoma was diagnosed a year later.

## Case presentation

**Figure 1:**
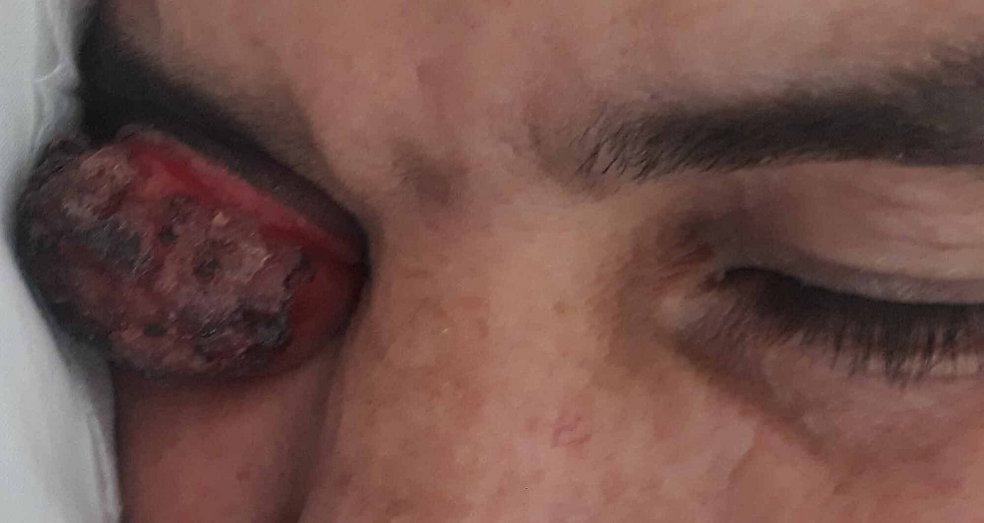
Clinical aspect of the ulcerated tumor.

We present the case of a 40-year-old man, who had a history of cavum UCNT (undifferentiated carcinoma of nasopharyngeal type) previously treated with Doxo-Cisplatine regimen, as well as radiation therapy, presenting to the emergency with a unilateral painless red eye. He had noticed painless eyelid redness in his right eye, as well as swelling which rapidly obstructed his vision. He described involuntary tear production, complete obstruction of vision in his right eye, but no purulent secretions. The patient reported loss of appetite as well as weight loss in the last three months. Upon examination, there was a ”salmon patch”, diffuse, raised, and rugged lesion of the right conjunctiva, which was completely obstructing the right eye (Figure 1). The mass was ulcerated and painless. Although cervical adenomegalies were present bilaterally, they were smaller in size than 1 cm.

**Figure 2:**
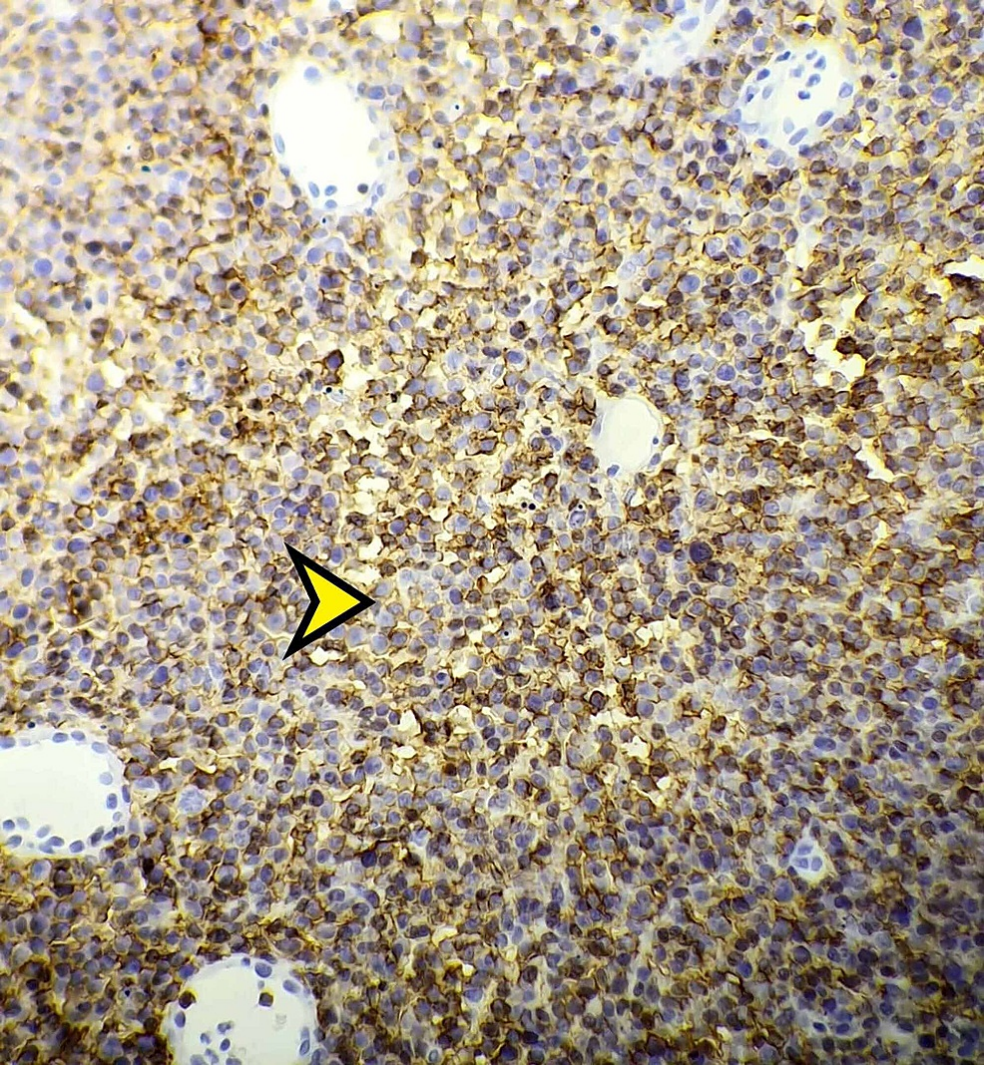
Diffuse anti-CD20 proliferation (yellow arrowhead) (original magnification x400).

**Figure 3:**
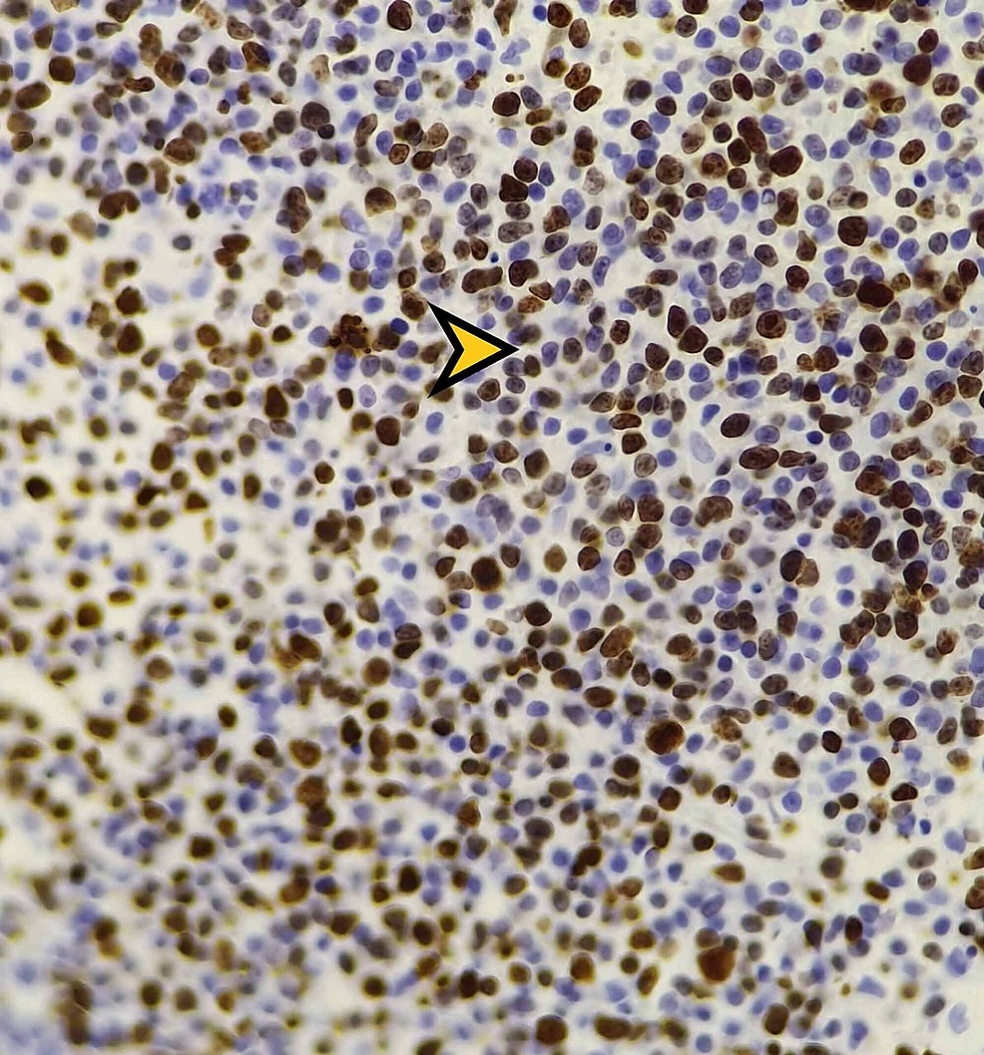
High ki-67 proliferative index (orange arrowhead) (original magnification x 400).

The patient underwent a conjunctival excisional biopsy. Pathological examination revealed diffuse proliferation of malignant lymphoid cells containing large prominent nucleoli with an abundance of mitotic figures. In addition, there was a diffuse positive staining with CD20 (Figure 2); the Bcl2, CD23, MUM1 stains were positive as well. We also observed a high Ki 67 proliferation index (Figure 3), consistent with a diagnosis of B-lineage non-Hodgkin’s lymphoma (NHL). The patient’s bone marrow biopsy and immunohistochemistry were normal.

Laboratory studies revealed a normal leukocyte count, as well as normal lactate dehydrogenase (LDH) and β2-microglobulin levels. The patient’s IPI (International Prognostic Index) score was zero. The result of his serum electrophoresis was also normal. His liver and kidney function tests were unremarkable. A CT scan of the chest, abdomen, and pelvis revealed no abnormalities. An orbital MRI scan confirmed the presence of a 30*13*25-millimeter conjunctival tumor, with no orbital involvement. There was no proof of UCNT involvement since the nasofibroscopy, as well as biopsies that were taken during it, were unremarkable.

**Figure 4:**
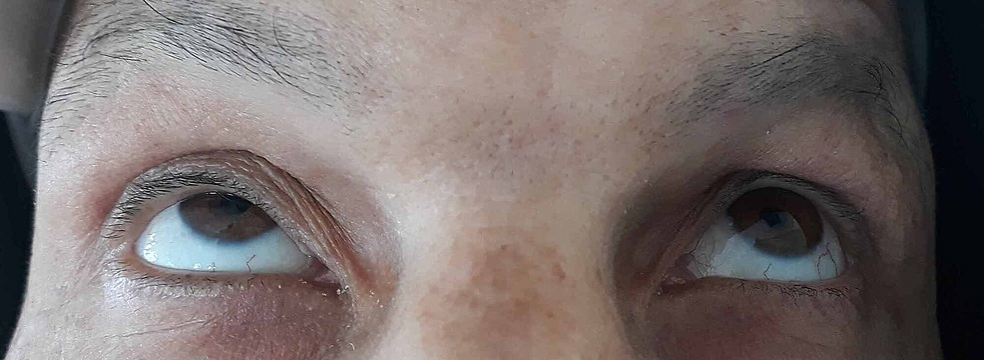
Complete regression of the tumor 2 months following the first chemotherapy cure.

These findings led to the staging of the disease: stage IE lymphoma according to the Ann Arbor staging system, and bT1BN0M0 according to the American Joint Comity on Cancer (AJCC) staging system. Given the history of the patient as well as the high risk of developing systemic disease, the optimal therapy was considered to be a combination of systemic chemotherapy and immunotherapy (R-CHOP: rituximab, cyclophosphamide, doxorubicin, vincristine, prednisone). Two months following the first treatment with chemotherapy, the conjunctival mass had massively regressed, leaving no ocular signs were left. The patient had normal orbit movement and his eyesight was not altered (Figure 4).

## Discussion

Lymphoma of the eyelid is a rare disease, its malignant subtype, diffuse large B-cell lymphoma (DLBCL) involving the conjunctiva, is a high-grade subtype. Its origin in most cases is primary and it constitutes 3% of conjunctival lymphomas [1]. Only a few patients are discovered to have a secondary lymphoma in comparison to the more aggressive subtype such as mantle cell lymphoma (MCL). To our knowledge, this is the first case of primary conjunctival diffuse large B-cell lymphoma occurring in a patient who previously had nasopharyngeal carcinoma. Given the aggressive type of DLBCL, chemotherapy is used as primary treatment, either as a single agent or as a combination regimen [2].

Our patient had a history of a previously treated malignancy and was thus considered to be at a high risk of developing systemic disease This guided the choice of immunotherapy with the R-CHOP regimen as a first-line treatment. Upon follow-up, the conjunctival lesion had completely disappeared, and the patient didn’t develop any sequelae. Finally, albeit conjunctival lymphoma appears to have a good prognosis, appropriate initial management, as well as long-term follow-up, are necessary because of the increased risk of developing systemic disease, and because of the existing risk of mortality [3].

Diffuse large B-cell lymphoma is associated with a 55% five-year survival [4].In the case of our patient, the early disease stage discovered at the time of his diagnosis correlates with a better long-term prognosis. Furthermore, the unilaterality of his tumor and its localized aspect at presentation are associated with a high survival rate [5].

## Conclusions

This case underlines the importance of being mindful of the development of primary lymphoma in the case of a patient with a history of treated carcinoma. Ocular involvement presenting as adnexal lymphoma must prompt an adequate histopathological diagnosis, as well as an assessment using adapted staging systems. These serve as the best indicator of prognosis, and therefore allow an optimization of the treatment.
